# Linear Copolymers Based on Choline Ionic Liquid Carrying Anti-Tuberculosis Drugs: Influence of Anion Type on Physicochemical Properties and Drug Release

**DOI:** 10.3390/ijms22010284

**Published:** 2020-12-30

**Authors:** Katarzyna Niesyto, Dorota Neugebauer

**Affiliations:** Department of Physical Chemistry and Technology of Polymers, Faculty of Chemistry, Silesian University of Technology, 44-100 Gliwice, Poland; Katarzyna.Niesyto@polsl.pl

**Keywords:** choline, anion exchange, antibacterial activity, polymer carriers

## Abstract

In this study, drug nanocarriers were designed using linear copolymers with different contents of cholinium-based ionic liquid units, i.e., [2-(methacryloyloxy)ethyl]trimethylammonium chloride (TMAMA/Cl: 25, 50, and 75 mol%). The amphiphilicity of the copolymers was evaluated on the basis of their critical micelle concentration (CMC = 0.055–0.079 mg/mL), and their hydrophilicities were determined by water contact angles (WCA = 17°–46°). The chloride anions in the polymer chain were involved in ionic exchange reactions to introduce pharmaceutical anions, i.e., *p*-aminosalicylate (PAS^−^), clavulanate (CLV^−^), piperacillin (PIP^−^), and fusidate (FUS^−^), which are established antibacterial agents for treating lung and respiratory diseases. The exchange reaction efficiency decreased in the following order: CLV^−^ > PAS^−^ > PIP^−^ >> FUS^−^. The hydrophilicity of the ionic drug conjugates was slightly reduced, as indicated by the increased WCA values. The major fraction of particles with sizes ~20 nm was detected in systems with at least 50% TMAMA carrying PAS or PIP. The influence of the drug character and carrier structure was also observed in the kinetic profiles of the release processes driven by the exchange with phosphate anions (0.5–6.4 μg/mL). The obtained polymer-drug ionic conjugates (especially that with PAS) are promising carriers with potential medical applications.

## 1. Introduction

Polymer-based drug delivery systems (DDS) are of great interest to the scientific community because of their various advantages. These vehicles improve the pharmacokinetics and pharmacodynamics involved in transporting the drug to the destination of therapeutic action [1,2,3,4]. Therefore, the most requested systems are the targeted and controlled DDS [5,6,7,8]. The delivery properties can be adjusted by modifying the polymer structure to avoid the uncontrolled rate of drug release [9,10,11]. Drug release can also be activated by specific conditions that are characteristic of unhealthy cells, i.e., pH, temperature, or ionic strength [12,13,14,15]. The responsiveness of polymer vehicles to pH changes are commonly used in the treatment of cancer cells due to their acidic reaction, which is an opposite to alkaline environment in healthy cells [16]. Additionally, the unhealthy cells usually gain higher temperature, which is exploited by temperature-responsive polymer carriers for specific behavior in the conditions of their lower or upper critical solution temperature [17]. Mechanisms based on ionic strength are characteristic for polymers containing ionic groups. Ion exchange controlled release systems are applied in delivery of ionic drugs, which can be attached to polymer matrix by ionic interactions. The ion delivery depends on the type and geometry of ionic carrier, charge intensity, and coordination number of ionic groups, as well as ionic strength of polymer solution [18].

Diverse types of carrier are known, some of them are polymer-drug conjugates, in which the latter component is connected directly to the polymeric matrix by a chemical bond or through the linker [19,20]. If the conjugate has amphiphilic properties, the drug can be encapsulated inside the self-assembled structure [21], thus constituting dual drug co-delivery systems, which have also been applied in combined therapies [22,23]. Compared with traditional drugs, the presence of a polymer carrier is intended to ensure the solubility of the hydrophobic drug in aqueous solution, which is desirable to optimize the drug’s effect on the human body [24,25]. Conjugate design requires individualized strategies that involve introducing the appropriate type of connection between the drug and the polymeric matrix to achieve the desired release profile [26,27].

Ionic liquids (ILs), which are popular in various fields of chemistry, are also convenient for drug delivery because of their ionic nature and ion exchange capabilities, which are useful to tune biochemical properties and to generate pharmaceutical activity. As a result, the drug (in an anionic or cationic form) can be introduced into polymer matrix, wherein it is ionically bonded with a counterion [28]. Choline ((2-hydroxyethyl)trimethylammonium chloride), as an IL with vitamin-like functions, is a known carrier of anti-inflammatory salicylates and can be applied as a biological cation owing to its biocompatibility and antibacterial properties [29,30,31]. Moreover, this molecule and its phosphoryl derivative are capable of degradation under anaerobic conditions [32,33]. The (phosphoryl)choline in a methacrylate-functionalized form has been polymerized to obtain a biocompatible poly(ionic liquid) (PIL) for pharmacological and medical applications. For example, poly(2-methacryloyloxyethyl) phosphorylcholine has been reported as a suitable component of implants, medical devices, or DDS [34,35,36]. Similarly, poly[2-(methacryloyloxy)ethyl]trimethylammonium chloride (PTMAMA) has demonstrated bactericidal and fungicidal properties [37]. Moreover, its high hydrophilicity can be modified by incorporating hydrophobic units into the polymer chain or by exchanging with hydrophobic anions to yield the amphiphilic copolymer [38], which can create micellar systems in aqueous solution and consequently enable encapsulation of non-ionic drugs [39]. On the other hand, the combination of quaternary ammonium cations with chloride counter ions has been convenient for anion exchange reactions to introduce the ionic drug [40], thus forming the ionic type of polymer-drug conjugates. These systems comprised the graft topology of PTMAMA carrying the pharmaceutical anions, such as salicylate [41], *p*-aminosalicylate, or clavulanate [42]. In these cases, the drug release occurred in the solution containing the stronger ions (i.e., phosphate ions), which is in contrast to conventional conjugates, where the drug is released by hydrolytic degradation of a linker (usually ester- or amide-type). Other PIL-based DDSs have been reported for poly(imidazolium salt)s carrying naproxen anions [43] or poly(guanidinium salt)s with antibiotic anions [44].

In the present work, we describe linear PILs based on [2-(methacryloyloxy)ethyl] trimethylammonium chloride (TMAMA) as the universal matrix for potential polymer-drug ionic conjugates. These copolymers with varied ionic group contents were synthesized via controlled polymerization reaction (i.e., atom transfer radical polymerization (ATRP)). New drug delivery systems were designed by employing the anion exchange reaction with selected drugs in ionic form, such as *p*-aminosalicylic (PAS^−^), clavulanic (CLV^−^), fusidic (FUS^−^), and piperacillin (PIP^−^) anions as the antibacterial agents. *p*-Aminosalicylic acid has antitubercular activity, which extinguishes the growth and multiplication of *Mycobacterium tuberculosis*, leading to cell apoptosis, so it is predominantly used to support the action of other anti-tuberculosis drugs. Clavulanic acid isolated from *Streptomyces* is a weak antimicrobial agent and β-lactamase inhibitor that precludes deactivation of antibiotic in combination therapy for bacterial infections (i.e., acute bronchitis and upper respiratory tract infections). Piperacillin acid as an ampicillin-derived antibiotic with broad-spectrum bactericidal activity, and it is clinically efficient in the treatment of infections caused by *Streptococcus pneumoniae*, including lung diseases. Fusidic acid is a natural steroid antibiotic that shows bacteriostatic activity without the corticosteroid effects, and it is effective against *Bordetella pertussis* and *Staphylococcus aureus*, which damage the respiratory system. The aforementioned drugs are typically administrated orally during conventional treatments. The influence of the carrier composition and the anion type on the physicochemical properties, exchange effect, and progress of pharmaceutics release are investigated in this work, in order to inform the design single-drug delivery systems with potential for the treatment of lung and respiratory diseases. Previously, PAS^−^ and CLV^−^ based systems have been studied in combination with PTMAMA graft copolymers [42]. However, in those cases the TMAMA units were located in the side chains, which were formed by grafting from polymethacrylate macroinitiator. Similarly to linear copolymers they were varied with content of ionic units, but in the grafted ones this range was narrower (13–46%), and the TMAMA copolymer side chains were significantly shorter (16–65 repeating units). Fundamental difference was the grafting degree (25% and 50%) defining the distribution density of side chains attached to the hydrophobic backbone. Thus, the linear TMAMA copolymers were investigated to evaluate their delivery potential in comparison to analogous ionic carriers with graft topology.

## 2. Results and Discussion

**Figure 1:**
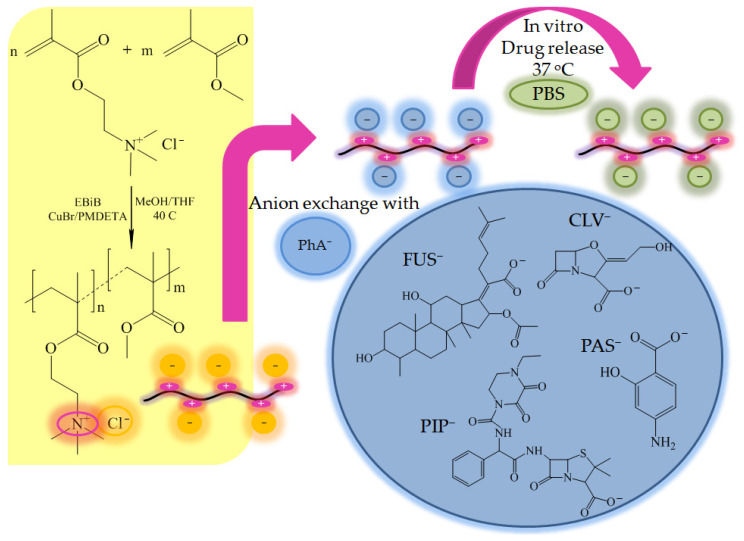
Schematic anion exchange pathway for obtaining linear TMAMA-based copolymers.

Several carriers were designed with statistically distributed ionic units, which act as polymer-drug conjugates. To obtain such systems, PILs containing an ionic monomer (i.e., TMAMA/Cl as M1) in combination with methyl methacrylate (MMA as M2) as a co-monomer were copolymerized in different ratios (C1: 25/75, C2: 50/50, and C3: 75/25) by ATRP at 40 °C. The reaction was initiated by ethyl 2-bromoisobutyrate (EBiB) and catalyzed by a copper bromide/*N*,*N*,*N*′,*N*″,*N*″-pentamethyldiethylenetriamine (CuBr/PMDETA) complex. As a result, linear copolymers were prepared with varying content of ionic units, which corresponded to the initial proportion of TMAMA/MMA in the reaction mixture (Figure 1, Table 1). In our previous studies on the copolymerization of TMAMA and MMA formation of statistical copolymers was postulated due to comparable relative reactivity ratios of comonomers, that is r_TMAMA/Cl_ = 1.13 and r_MMA_ = 0.88 [38].

The use of methanol as co-solvent in the reaction mixture can be disadvantageous because of possible transesterification of TMAMA/Cl to MMA as side reaction during polymerization [45,46]. Our previous studies showed that it can be minimized effectively at reduced amount of methanol (1 mL per 1 g of TMAMA) and at higher initial content of TMAMA (≥25%) [38]. Thus the use of optimized conditions of copolymerization, i.e., ratios of TMAMA/MMA (25/75, 50/50, 75/25), and low amount of methanol in the reaction mixture (1 mL per 1 g of TMAMA), allowed excluding transesterification, which was confirmed by the calculated content of ionic fraction (F_MI_) in the copolymer (it was different from that initial by 1%).

**Figure 2:**
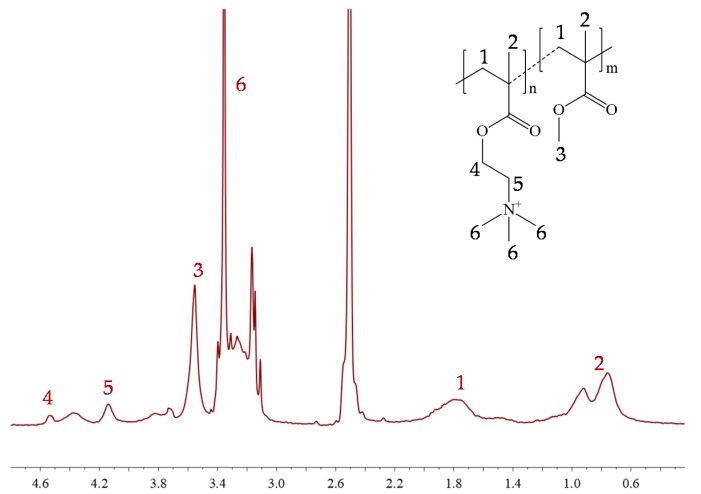
^1^H NMR spectrum of linear copolymer of TMAMA/Cl C1, where n is DP_M1_ and m is DP_M2_.

The copolymer structure was confirmed by proton nuclear magnetic resonance (^1^H NMR) spectroscopy, which revealed proton signals from methyl groups at 1.4–0.6 ppm and methylene groups at 2.0–1.6 ppm in the main chain (Figure 2). Additionally, signals from methoxy protons at 3.7–3.5 ppm in MMA units, and from oxyethylene and methyl groups in the ammonium cation of TMAMA/Cl units (4H at 4.6–4.1 ppm and 9H groups at 3.4 and 3.3 ppm, respectively) confirmed the incorporation of both monomers into the polymer chain. The ^1^H NMR analysis of the reaction mixture allowed for the determination of monomer conversions (Table 1) using the integration of vinyl proton signals assigned to unreacted TMAMA and MMA (6.2–6.1 ppm and 6.1–6.0 ppm, respectively) relative to signals corresponding to the protons in substituents (both reacted and unreacted monomers), i.e., 9H in -(CH_3_)_3_N^+^ and 3H in -OCH_3_, respectively. The conversion values could then be used to calculate the other parameters, including the polymerization degree, the ionic unit contents, and the polymer molecular weight (Table 1). The compositions of the final copolymers C1, C2, and C3 containing 24%, 49%, and 75% TMAMA units, respectively, were guaranteed by properly assumed initial ratio of monomers. The controlled polymerization was also verified by the size-exclusion chromatography (SEC) results, supporting low dispersity indices of the polymers with longer chains (Ð < 1.4 at DP_n_ > 450). The exception for C1 suggests that a lower reaction rate in a system containing predominantly MMA promoted the occurrence of side reactions (Table 1).

**Figure 3:**
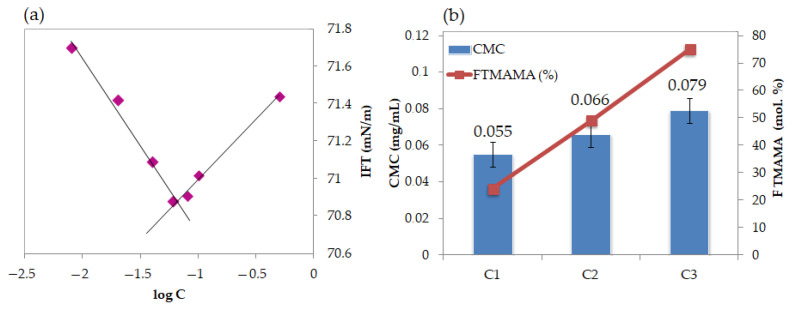
(**a**) Variation in the interfacial tension with the logarithm of the linear copolymer C2 concentration in aqueous solution at 25 °C; (**b**) influence of polymer chain length on critical micelle concentration (CMC) value.

The amphiphilic nature of the linear copolymers was investigated via goniometry; specifically, measuring the interfacial tension (IFT) of polymer aqueous solution in a concentration series (C = 0.008–0.5 mg/mL). The crossing point on a plot of IFT values vs. log C of measured samples was determined as the critical micelle concentration (CMC; Figure 3a), which defines the polymer’s ability to form micellar structures in aqueous solution. The CMC values increased as the content of TMAMA ionic units increased (Figure 3b).

The presence of cationic units with chloride counter ions statistically distributed along the polymer chain was advantageous for the anion exchange reaction to generate pharmaceutical activity. The drug in ionic form was introduced to the polymer matrix by dissolving and mixing with the polymer for 48 h. The studied pharmaceutical salts (i.e., sodium or potassium PAS, CLV, PIP, and FUS) were selected based on their antibacterial and bacteriostatic activity. The introduction of pharmaceutical anion (PhA) into chloride based copolymers was analyzed by Fourier-transform infrared spectroscopy (FT-IR), where the characteristic bands were identified. Before anion exchange the chloride copolymers were recognized by the presence of absorption peaks corresponding to C-O and C=O stretching vibration of ester groups (1150 cm^−1^ and 1720 cm^−1^, respectively), C-H (1450 cm^−1^, 2800–3100 cm^−1^), as well as C-N (950 cm^−1^) in the quaternary nitrogen group of TMAMA units (Figure S1). After anion exchange the new bands appeared at 800–1000 cm^−1^ and 1600 cm^−1^ (aromatic C=C) due to PAS and PIP, at 1550 cm^−1^ (alkene C=C) due to CLV and FUS, 1250 cm^−1^ (C-S) due to PIP. Moreover, a broad signal characteristic for O-H and N-H stretching vibrations (3250–3600 cm^−1^) was detected as the representative for all types of anions, whereas signal in range of 2845–3000 cm^−1^ was significantly more intense in the system with CLV due to content of large number of aliphatic and cyclic C-H bonding.

A new characteristic signals assigned to the bonded drugs were also detected in the ^1^H NMR spectra (see detailed data of chemical shifts in the Supporting Materials). The protons becoming from the polymer matrix are observed at low ppm region (4.58–0.46 ppm, Figure S2a). After anion exchange to PAS^−^ the conjugation was confirmed by the presence of signals in the range of 4.9–7.3 ppm, including protons from benzene ring (Figure S2b). In the case of CLV, PIP, and FUS (Figure S2c–e), the protons corresponding to PhA were overlapped with those for the copolymer carrier. Theoretically, the pharmaceutical anions could generate the nucleophilic attack on the carbon of carbonyl group to form carboxylic groups in the copolymer. However, both carboxylic acids and their deprotonated forms are rather weak nucleophiles, thus they should not be effective in the case of the detachment of methyl and 2-trimethylammonium ethyl groups situated at ester bonding. The lack of signal at higher chemical shifts (12–13 ppm) in the ^1^H NMR spectra of the ionic conjugates confirms that the acidic hydrolysis was not activated (Figure S2).

**Figure 4:**
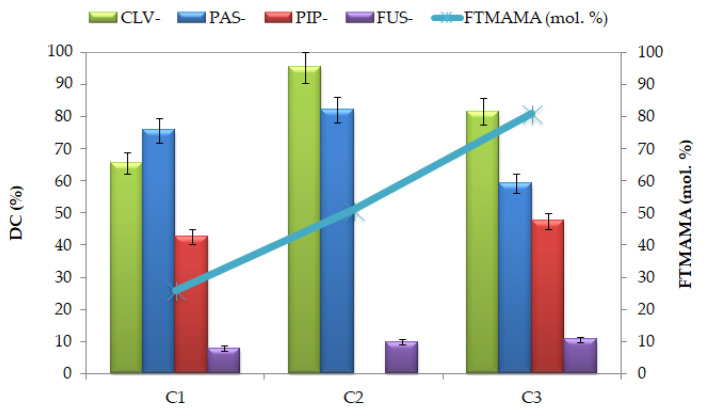
Effect of anion type and content of ionic fractions on the drug content.

The degree of anion exchange was determined based on the drug content (DC), which was evaluated with ultraviolet-visible light spectroscopy (UV-Vis). DC values represent the percentage contribution of pharmaceutical ions in the copolymer (Table 2). In the exchange reactions, the least effective ionic drug was FUS (7–11%), but CLV, PAS, and PIP, demonstrated satisfactory degrees of exchange, reaching levels of 65–95%, 59–82%, and 42–48%, respectively. These results suggest that the nature of the anion has a significant impact on the degree of exchange, and that pharmaceutic compounds with less steric hindrance (i.e., PAS and CLV) were especially beneficial in the anion replacement reaction. Additionally, the drug content was improved when the TMAMA content was increased to 50%, but a larger quantity of ionic units limited the exchange yield (Figure 4). The PIP and FUS anions may have encountered problems finding the chloride exchange sites because they are larger molecules. The structures of PAS, CLV, and PIP contain the nitrogen atoms, which provide their higher coordination number, and they enhance the anion affinity to trimethylammonium substituents. As a result, these pharmaceutical anions were introduced in large amounts. A different effect was noticed in the case of FUS, which is a rigid structure of four conjugated rings, thus exhibiting lower affinity for the hydrophilic moieties. These results are in good correlation with another important factor, which is the water solubility of the salt/anion, which is the most limited for FUS, and then increases in the order PIP < PAS < CLV, according to Databank Online.

**Figure 5:**
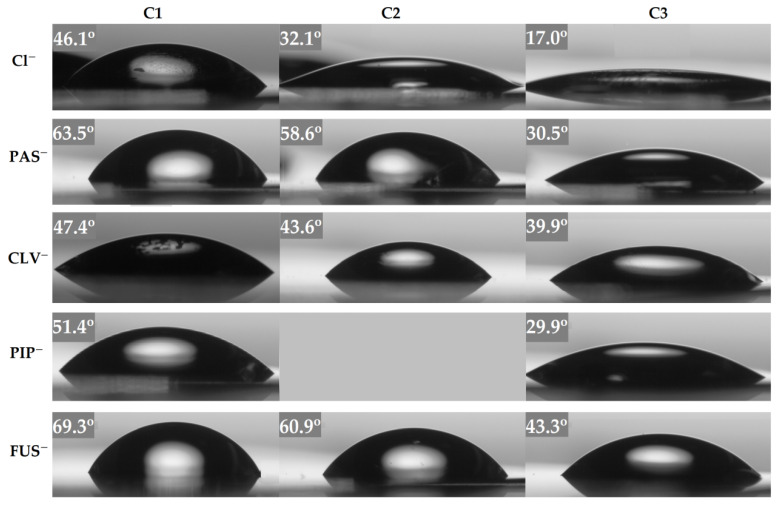
Changes in surface wettability depending on the copolymer and counter-ion, illustrated by goniometry camera.

The hydrophilicities of the introduced pharmaceutical anions were evaluated by determining the water contact angle via goniometry using the sessile drop method. Aqueous solutions of the polymer systems (0.3 mg/mL in methanol) were applied to thoroughly cleaned glass plates by a spin-coating method, where the centrifugal force allowed for the homogeneous distribution of polymer across the surface to form a film. The water contact angles (WCA) values (presented in Figure 5) showed a decreasing trend with increasing content of ionic units (F_M1_) for all studied systems, including those with the pharmaceutical anions. 

The WCA values for polymer matrices containing Cl anions changed from 46° to 17°, indicating that they were hydrophilic systems. The same correlation between WCA and TMAMA content has been described previously for graft copolymers with TMAMA units in the side chains [42], but their wettability was lower than that of the linear copolymers studied herein because of the hydrophobic backbone. After anion exchange, the wettability of the polymer films was reduced, yielding higher WCA values relative to their polymer matrices, meaning that the selected drugs did not improve the hydrophilicity of ionic conjugates. Among the tested systems, the FUS-bearing polymers were the most dissimilar (43–69°), although the drug content was the smallest, indicating the specificity of the steroid structure of the introduced anions. Figure 5 shows the visual changes in the contact angles of the individual systems, which illustrate the differences in the wettability of surfaces covered with PIL layers. The composition of the polymer matrix, the type of drug, and the exchange efficiency influence the hydrophilicity of the system. These parameters are also responsible for the ratios of TMAMA/PhA to TMAMA/Cl units present in the polymer chain due to incomplete exchange.

**Figure 6:**
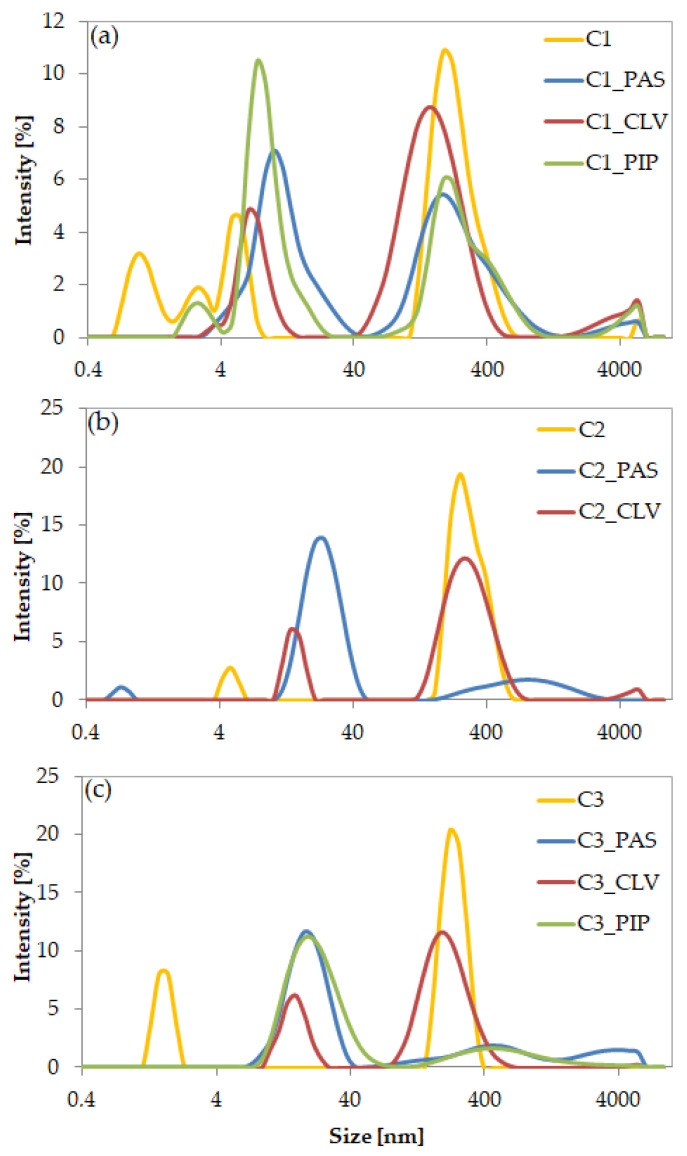
DLS histograms for particles of (**a**) C1, (**b**) C2, and (**c**) C3 systems in deionized water at 25 °C.

The physicochemical characteristics of the chloride-based copolymers and their ionic drug conjugates were evaluated using dynamic light scattering (DLS) measurements. Before exchange, the hydrodynamic diameters (D_h_) of copolymer particles were ranged in 240–300 nm showing no dependency on the ionic content. Copolymer C1 containing PAS and PIP showed similar behavior, forming two equal-volume fractions, with particle sizes of 274 and 12 nm, 291 and 9 nm, respectively. This result indicates that the self-assembled micelles easily aggregated because of the high content of hydrophobic fraction in C1 (F_MMA_ = 75%). In contrast, C2 and C3 the fraction of smaller particles (D_h_ ~20 nm) prevailed (67–80%). Additionally, the particles were characterized by a low size distribution (PDI = 0.38–0.46). In the copolymers with CLV, one dominant fraction was observed (75–80%), wherein the particles reached larger sizes (169–306 nm) and their polydispersity indices were higher (0.8–1). However, C1/CLV particles were smaller than C1 conjugated with PAS or PIP. The formation of large particles as the higher level of self-assemblies was observed due to aggregation effect of micelles in water solution. The self-organized linear TMAMA copolymers probably form the superstructures of entangled chains with statistically distributed ionic and hydrophobic groups, which can be situated in their outer part. Thus, the external hydrophobic moieties participating in the *π*-stacking interaction and hydrogen bonding between hydrophilic moieties (especially in the conjugates), were responsible for attraction polymer assemblies to result in aggregates (>470 nm, 20%) and super-aggregates (>1000 nm, <10%). The details are presented in Table 3 and illustrated by the DLS histograms in Figure 6.

The release of the selected drugs in non-ionic forms has been reported for the non-ionic polymers used in the encapsulation of these compounds via physical interactions, for example: poly(ε-caprolactone)-*b*-polyethylene glycol-*b*-poly(ε-caprolactone) polymersomes with loaded clavulanic acid (16%), which was released in 35% after 24 h and 60% after 170 h [47], poly(ethylene glycol) methyl ether-*b*-poly (lactide-*co*-glycolide) with loaded PIP/tazobactam to design the effective antibiofilm [48], poly(DL-lactic-*co*-glycolic acid) and poly(3-hydroxybutyric acid-*co*-3-hydroxyvaleric acid) microspheres with encapsulated fusidic acid (76–89%), which was released up to 80% [49].

Previous in vitro studies into the mechanism of anionic drug release from ionic carriers have indicated that the phosphate anions from phosphate buffered saline (PBS) can exchange with the biologically active anions conjugated to the polymer matrix [36]. Systems based on TMAMA copolymers have been investigated for delivery of salicylate or sulfacetamide counterions [39]. As it was noticed, the anion exchange properties strongly depended on the nature of both ionic polymer and ionic drug. Thus, the chloride anions were exchanged by the salicylate ones in 50%, and then they were exchanged by phosphates anions in 35–60% demonstrating the “burst” effect within 4 h, whereas in the case of sulfacetamide systems PhA introduction was efficient in 98%, but its release occurred in only 11%.

**Figure 7:**
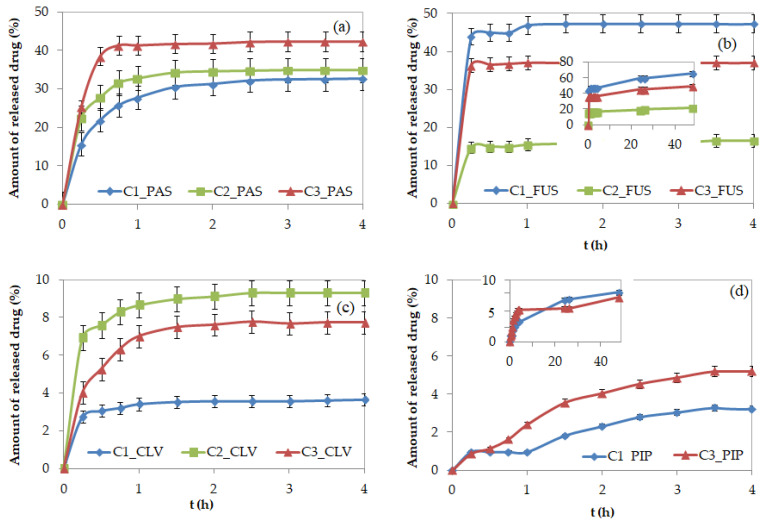
Kinetic release profiles of (**a**) PAS, (**b**) FUS, (**c**) CLV, and (**d**) PIP anions from conjugates based on PILs.

During drug release via dialysis in PBS (pH = 7.4 in 37 °C), the samples were collected at designated time intervals, and the maximum absorptions of the free pharmaceutics were measured using UV-Vis. The burst release of PAS^−^ and CLV^−^ was observed around 1–1.5 h, and the release continued up to 4 h, when small changes in the kinetics profiles resulted in the plateau state. For the systems containing PIP^−^ and FUS^−^, which have large steric hindrance, the release process occurred over a longer time, as indicated by the visible changes in free drug concentration detected for up to 48 h, especially for PIP systems (Figure 7). Generally, four different release profiles depending on the burst drug release point and more or less suddenly attained plateau are demonstrated. According to that the PAS, FUS, and CLV based systems in comparison to polymers bearing PIP seem to be more effective in the release rate of those anions. The rapid release of the drug in the first 4 h suggests their localization in the external groups, whereas the remaining anions could be trapped inside. This hypothesis can be concluded especially for PIP systems, where the effective drug release followed after 1 h. The remaining drug anions trapped inside the core need higher ionic strength to suppress interactions and then to diffuse though the entangled matrix, which can be additionally limited by the rigidity and steric hindrance of pharmaceutic molecule. The best delivery properties considering both drug content and amount of the released drug were exhibited by PAS systems, because 60–80% of the drug was attached and 33–46% was released. This was associated with a high concentration of released drug (3.0–6.4 μg/mL; Table 2). Although the systems with FUS^−^ could exchange large amounts of pharmaceutics with phosphate anions (21–66%) probably due to repulsive effect magnified by steric structure of FUS, the low drug content (~2 μg/mL) led to a significantly lower concentration of the released drug (0.5–1.2 μg/mL). Low release percentage levels were achieved by the systems containing CLV^−^ and PIP^−^ (~10%) corresponding to 0.8–2.0 mg/mL, in spite of a relatively large drug contents in CLV^−^ and PIP^−^ systems (mean ~80% vs. 40%). This opposite behavior in relation to FUS can be also explained by the way of anions conjugation and high stability of conjugates due to strong attraction in entangled core and lower coordination number of phosphate anions.

The previous tested micelles based on TMAMA grafted copolymers formed micelles, where the core was composed mainly of hydrophobic backbone; in turn, the shell included grafted chains with cationic groups. Thus, the pharmaceutical anions were arranged only outside the core. In comparison with linear copolymers, the easier access to the pharmaceutical ions in grafted ones should facilitate drug release, but this aspect is much more composite due to higher stability of the graft polymer with the micelle-like structure. The exchange of Cl^−^ onto PAS^−^ and CLV^−^ in the graft polymer matrix was yielded at 31–64% and 79–100%, respectively, and then the exchange onto phosphate anions was carried out during drug release in 20–42% (3–9 µg/mL from 1 mg of drug conjugates) for PAS and 25–73% (11–31 µg/mL) for CLV within 3 h [42]. These results indicated similar levels of PAS release independently on polymer topology, whereas the percentage amounts of released CLV was ~10 times lower for the linear polymers. The burst effect was much less significant for graft copolymers, especially those with higher grafting degree. It shows that the change in structural parameters of copolymer carrier caused by macromolecule topology can be strategic for individual exchange and release properties for the same pharmaceutical anion.

**Figure 8:**
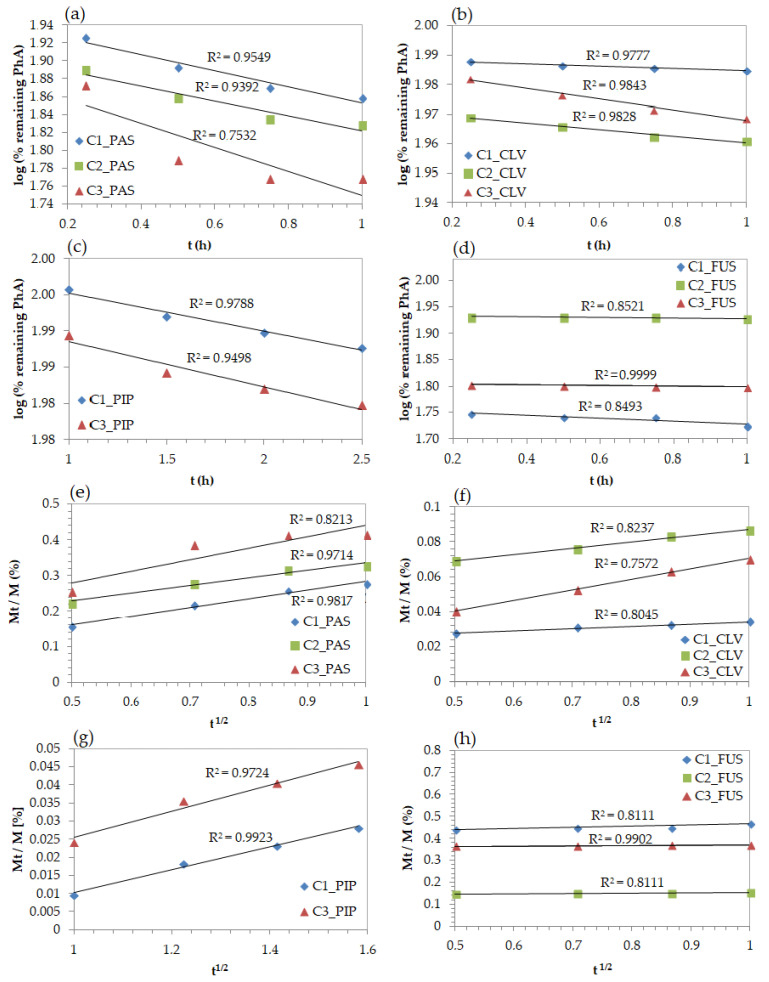
Kinetics profiles by models of first order (**a**–**d**), and Higuchi’s (**e**–**h**) for release of PAS, CLV, PIP, and FUS anions from conjugates based on PILs.

Different mathematical models were applied to describe the in vitro drug release. The fitting levels for kinetics profiles were interpreted by correlation coefficients (R^2^), which determined adequate model describing the release mechanisms. The release of PAS, CLV, PIP, and FUS anions represented by first-order kinetics (R^2^ = 0.75–0.95, 0.98, 0.95–0.97, 0.84–0.99, respectively) indicated the dependence of drug release rate on the concentration (Figure 8a–d). The Higuchi model, presented as a function of the percentage of remaining anions in relation to the square root of time (Figure 8e–h), was the most fitted for PIP conjugates showing the highest values of the R^2^ above 0.97. However, the CLV and FUS systems also achieved good correlation with this model (R^2^ = 0.81–0.99). Hence, it can be postulated that the anion drug release followed diffusion controlled mechanism. Moreover, it was found that the Korsmeyer–Peppas model, which distinguishes Fickian and non-Fickian diffusion estimated by the diffusion exponent (*n*, calculated from equation M_t_/M = *kt^n^*, Table S1) similarly to Higuchi model, was also represented by a high fitting degree (0.8–0.99; Figure S3). System C3/PIP indicated the diffusion exponent value in the range of equal 0.45 < *n* < 0.89, so the drug release occurred by non-Fickian diffusion, whereas in the case of C1/PIP it was higher than 0.89, which implies non-Fickian super case-II transport. The release of PhA by other systems can be described by quasi-Fickian process (*n* ≤ 0.45). These results mean that the diffusion process of released anions is slower than the anion exchange as consequence of the break of ionic bonding between PhA and trimethylammonium moieties in the polymer.

## 3. Materials and Methods

The methyl methacrylate (MMA; Alfa Aesar, Warsaw, Poland) was dried using molecular sieves (type 4Å, Chempur, Piekary Śląskie, Poland). The [2-(methacryloyloxy)ethyl]-trimethylammonium chloride (TMAMA/Cl; 80% aq. solution, Sigma-Aldrich, Poznań, Poland) was dried to a constant weight under reduced pressure. Methanol and tetrahydrofuran (THF) were purchased from Chempur (Piekary Śląskie, Poland) and dried over the same molecular sieves as MMA. Copper(I) bromide (CuBr; Fluka, 98%, Steinheim, Germany) was purified using a procedure described previously in the literature [42]. *N,N,N′,N*″*,N*″-pentamethyldiethylenetriamine (PMDETA, 98%), ethyl 2-bromoisobutyrate (EBiB, 98%), potassium clavulanate (KCLV), and bis(trifluoromethane)sulfonimide lithium salt (LiTf_2_N) were obtained from Sigma Aldrich (Poznań, Polska). Sodium *p*-aminosalicylate (NaPAS, 98%), sodium piperacillin (NaPIP, 99%), and sodium fusidate (NaFUS, 98.8%) were purchased from Alfa Aesar (Warsaw, Poland) and used without further purification.

### 3.1. Characterization

^1^H-NMR spectra were recorded using a UNITY/NOVA spectrometer (300 MHz, Varian, Mulgrave, Victoria, Australia). The measurements were executed in deuterated dimethyl sulfoxide (DMSO-d_6_) with tetramethylsilane (TMS) as the internal standard. The molecular weight (M_n_) and dispersity index (Ð) were estimated using size exclusion chromatography (SEC) in THF (1100 Agilent 1260 Infinity with differential refractometer MDS RI detector, Agilent Technologies, Santa Clara, CA, USA) at 40 °C with a flow rate of 0.8 mL/min using a pre-column guard (5 × 7.5 mm) and a PLGel 5 μm MIXED-C 300 column (7.5 × 300 mm). The calculations were based on polystyrene standards (580–300,000 g/mol). Fourier-transform infrared spectroscopy (FT-IR) was conducted with Spectrum Two 1000 FT-IR Infrared Spectrometer with attenuated total reflection (ATR) (Perkin Elmer, Waltham, MA, USA). The critical micelle concentration (CMC) was determined via interfacial tension measurements of aqueous polymer solutions with concentrations in the range of 8 × 10^−3^ to 0.5 mg/mL, which were performed with the pendant drop method using a goniometer (OCA 15EC, DataPhysics, Filderstadt, Germany). SCA20_U software was used for data collection and processing. The same software module was also employed for the water contact angle (WCA) measurements carried by the sessile drop method, in which a drop of water (4 μL) was placed on the polymer film. The polymer film was prepared by spin coating the polymer solution in methanol (0.3 mg/mL), which was applied on degreased glass plates. The hydrodynamic diameter (D_h_) of particles and polydispersity index (PDI) were measured by dynamic light scattering (DLS) using a Zetasizer Nano-S90 instrument (Malvern Technologies, Malvern, UK). He-Ne laser operated at 4 mW was the source of light scattered at 633 nm. Samples diluted in water (1.0 mg/mL) after filtration (MCE Syringe Filters, hydrophilic M.E. Cellulose membrane with pore diameter: 0.45 μm) were introduced into poly(methyl methacrylate) cells, which were placed in the compartment thermostated at 25 °C. Each measurement was repeated three times to obtain an average value. Data were analyzed using the cumulant method. The drug release process was monitored by ultraviolet-visible spectroscopy (UV-Vis; Evolution 300 spectrometer, Thermo Fisher Scientific, Waltham, MA, USA) on the samples acquired at suitable time intervals. This method allowed the determination of the drug content (DC) and the amount of released pharmaceutical anions (PAS^−^, CLV^−^, PIP^−^, or FUS^−^).

### 3.2. Synthesis of Linear Copolymers Bearing Cl^−^ (Example for C1)

Comonomers TMAMA (2 g, 9.63 mmol) and MMA (3.08 mL, 28.88 mmol), methanol (3 mL), and THF (1 mL) were added into a Schlenk flask. The mixture was degassed by two freeze-pump-thaw cycles. Then, EBiB (9.52 μL, 0.06 mmol) was added as the ATRP initiator, and the degassing cycle was repeated once again before the initial sample (0.1 mL) was taken. The reaction was catalyzed by a CuBr/PMDETA complex (9.21 mg, 0.06 mmol/13.40 μL, 0.06 mmol). The reaction ran for 24 h at 40 °C and was stopped by exposing the mixture to the air. The polymer was dissolved in methanol and precipitated twice in a chloroform-diethyl ether mixture, then dried under vacuum. ^1^H-NMR (300 MHz, DMSO-d_6_, ppm): 4.58–4.50 (2H, -O-CH_2_-), 4.21–4.08 (2H, -CH_2_-N^+^), 3.66–3.46 (3H, -OCH_3_), 3.42–3.31 (9H, -N^+^(CH_3_)_3_), 2.09–1.60 (2H, -CH_2_-), 1.40–0.46 (3H, -CH_3_).

### 3.3. Anion Exchange Reaction in Polymer Matrix (Example for C1)

The copolymer C1 (28.11 mg, including 0.05 mmol of ionic units) was dissolved in methanol (1 mL) and mixed with the proper pharmaceutical salt (i.e., NaPAS (11.70 mg, 0.06 mmol), KCLV (11.36 mg, 0.06 mmol), NaFUS (29.86 mg, 0.06 mmol), or NaPIP (29.91 mg, 0.06 mmol). The reaction was carried out for 48 h. The ionic conjugates with pharmaceutical anions (PhA) were obtained after drying under reduced pressure.

To analyze the molecular weight and dispersity index of the synthesized chloride-containing copolymers (insoluble in standard SEC solvents, including THF), the ion exchange of chloride anions with bistriflimide ions was performed. Copolymer C1 (3.82 mg, including 0.01 mmol of ionic units) was dissolved in methanol, and then, LiTf_2_N salt (2.16 mg, 0.01 mmol) was added. The polymer with exchanged anions (Tf_2_N^−^) was dried under reduced pressure to give the final product.

### 3.4. Drug Release of Pharmaceutical Anions

The conjugate with pharmaceutical anions (1.0 mg) was dissolved in 1 mL of phosphate buffered saline (PBS) aqueous solution (pH = 7.4). Then, 1 mL of polymer solution was placed into a dialysis membrane bag (MWCO = 3.5 kDa) and put into a glass vial filled with 45 mL PBS. Then, the solution was stirred at 37 °C for 48 h. During the drug release, buffer samples (2.5 mL) were collected at consistent time intervals and analyzed on a UV-Vis spectrophotometer. The absorbance of PAS, CLV, FUS, or PIP was measured at 261, 258, 256, or 260 nm, respectively. The quantitative content of the drug in PBS was calculated based on the prepared calibration curve and appointed absorbance maximum wavelengths for the anions with antibacterial activity.

## 4. Conclusions

Several choline-based copolymers were synthesized by ATRP with different content of ionic units (i.e., 25%, 50%, and 75%), with the ability for self-assembly at CMC below 0.08 mg/mL. The presence of trimethylammonium groups and chloride anions in the main polymer chain was convenient for introducing various pharmaceutical anions with antibacterial properties via an exchange reaction. The resulting conjugates, which act as nanocarriers of ionic drugs, could be applied as oral medicines, owing to their suitable particle sizes (19–306 nm). The conjugated drugs did not exhibit significantly different levels of hydrophilicity in comparison with their chloride matrices, which was confirmed by the wettability of the polymer films evaluated by WCA (the maximum increment was 25°). The developed systems were capable of effectively carrying PAS, CLV, and PIP anions, with contents of 59–82%, 66–95%, and 43–47%, respectively. During the release process, PAS anions were removed by phosphate ions from the polymer systems in the largest amounts (33–46%; 3–6 μg/mL). The specific attractive force of CLV and PIP anions to the polymer matrix likely inhibited the release of the drug, and they were replaced at ~10%. The low content of FUS anions induced significant release (21–66%). The designed ionic conjugates carrying pharmaceutical anions with antibacterial activity may represent interesting systems with potential applications for lung and respiratory treatment, especially because of their physicochemical characteristics and preliminary drug delivery results.

## Figures and Tables

**Table 1:** Characteristics of linear copolymers based on choline ionic liquid (IL) synthetized by atom transfer radical polymerization (ATRP).

		^1^H NMR ^a^						SEC ^b^	
No.	M1/M2	X_M1_ (%)	X_M2_ (%)	DP_M1_	DP_n_	F_M1_ (%)	M_n_ × 10^3^ (g/mol)	M_n_ × 10^3^ (g/mol)	Ð
C1	25/75	59.8	62.3	90	370	24	46.9	5.7	1.74
C2	50/50	78.6	80.5	236	477	49	73.3	11.9	1.36
C3	75/25	88.1	86.6	396	526	75	95.5	16.7	1.27

M1: TMAMA/Cl, M2: MMA; conditions: C1: [M1+M2]_0_:[EBiB]_0_:[CuBr]_0_:[PMDETA]_0_ = 600:1:1:1, 24 h, methanol:THF = 3:1 *v/v*, where methanol:M1 = 1:1 *v/wt*., 40 °C; X_M1_ and X_M2_ are conversions of TMAMA/Cl and MMA, respectively; DP_M1_ is polymerization degree of TMAMA/Cl units; DP_n_ is polymerization degree; F_M1_ is content of TMAMA/Cl fraction in the polymer. ^a^ deuterated dimethyl sulfoxide (DMSO-d_6_), tetramethylsilane (TMS) internal standard; ^b^ tetrahydrofuran (THF) solvent, polystyrene calibration.

**Table 2:** Data for exchanged and released pharmaceutical anion (PhA) in the TMAMA-based polymers.

	Drug Exchange				Drug Release			
	DC (%) (Concentration of Introduced PhA (μg/mL))				Amount of Released PhA (%) (Concentration of Released PhA (μg/mL))			
No.	PAS^−^	CLV^−^	PIP^−^	FUS^−^	PAS^−^	CLV^−^	PIP^−^	FUS^−^
C1	75.8	65.6	42.6	7.8	32.7	5.3	8.1	65.7
	(16.9)	(14.6)	(9.5)	(1.7)	(5.3)	(0.8)	(0.8)	(1.1)
C2	82.2	95.3	-	9.7	34.9	9.3	-	21.1
	(18.2)	(21.2)		(2.2)	(6.4)	(2.0)		(0.5)
C3	59.3	81.7	47.5	10.7	45.4	7.7	7.3	49.5
	(6.6)	(18.1)	(10.5)	(2.4)	(3.0)	(1.4)	(0.8)	(1.2)

DC is drug content; PAS^−^ is *p*-aminosalicylic, CLV^−^ is clavulanic, FUS^−^ is fusidic, and PIP^−^ is piperacillin anion.

**Table 3:** Hydrodynamic diameters of poly(ionic liquid) (PIL) particles determined by dynamic light scattering (DLS) ^a^.

No.	Cl^−^			PAS^−^			CLV^−^			PIP^−^		
	D_h_ (nm)	f (%)	PDI	D_h_ (nm)	f (%)	PDI	D_h_ (nm)	f (%)	PDI	D_h_ (nm)	f (%)	PDI
C1	239	62.7	0.723	274	49.9	0.605	169	73.4	0.809	291	43.7	0.496
	1–5	35.6		12	47.4		7	20.8		9	48.0	
C2	301	93.9	0.471	24	75.3	0.442	306	80.9	1.000	-	-	-
	5	6.1		926	22.1		15	17.0				
C3	237	76.3	0.506	20	67.2	0.457	217	75.6	0.791	23	80.5	0.381
	2	23.7		475	20.6		16	23.8		772	19.5	

Where f is an fraction content of particles, ^a^ measurements based on the intensity at a copolymer concentration in aqueous solution of 1 mg/mL.

## Data Availability

Not applicable.

## Supplementary Materials

The following are available online at https://www.mdpi.com/1422-0067/22/1/284/s1.

## Abbreviations

ATRP	Atom transfer radical polymerization
CLV	Clavulanate
CMC	Critical micelle concentration
DC	Drug content
DDS	Drug delivery system
DLS	Dynamic light scattering
DMSO-d_6_	Deuterated dimethyl sulfoxide
EBiB	Ethyl 2-bromoisobutyrate
FUS	Fusidate
IFT	Interfacial tension
LiTf_2_N	Bis(trifluoromethane)sulfonimide lithium salt
MMA	Methyl methacrylate
NMR	Nuclear magnetic resonance
PAS	*p*-Aminosalicylate
PBS	Phosphate buffered saline
PDI	Polydispersity index
PhA	Pharmaceutical anion
PIL	Poly(ionic liquid)
PIP^−^	Piperacillin
PMDETA	*N,N,N′,N*″*,N*″-pentamethyldiethylenetriamine
SEC	Size exclusion chromatography
THF	Tetrahydrofuran
TMAMA/Cl	[2-(methacryloyloxy)ethyl]trimethylammonium chloride
TMS	Tetramethylsilane
UV-Vis	Ultraviolet-visible light spectroscopy
WCA	Water contact angle
