# Supplementary material for: Linear Copolymers Based on Choline Ionic Liquid Carrying Anti-Tuberculosis Drugs: Influence of Anion Type on Physicochemical Properties and Drug Release
Source: Int J Mol Sci. 2020 Dec 30;22(1):284. doi: 10.3390/ijms22010284 (PMC7795545; doi:10.3390/ijms22010284)
Supplement: Supplementary file 1 [file ijms-22-00284-s001.pdf]

Supplementary Materials

Linear copolymers based on choline ionic liquid carrying anti-tuberculosis drugs: influence of anion type on physicochemical properties and drug release

Katarzyna Niesyto [1], Dorota Neugebauer [1*]

[1] Department of Physical Chemistry and Technology of Polymers, Faculty of Chemistry, Silesian University of Technology, 44-100 Gliwice, Poland
* Correspondence: Dorota.Neugebauer@polsl.pl

Content:

Figure S1. FT-IR spectra for C1 copolymer and its conjugates with PAS, CLV, PIP and FUS.

Figure S2. ^{1}H NMR spectra of (a) linear copolymer C1 vs (b) PAS, (c) CLV, (d) PIP and (e) FUS contained conjugates.

Figure S3. Kinetics profiles by Korsmeyer-Peppas model for release of (a) PAS, (b) CLV, (c) PIP, and (d) FUS anions from conjugates based on PILs.

Table S1. Fitting and diffusion coefficients of drug release kinetics by Korsmeyer-Peppas model.

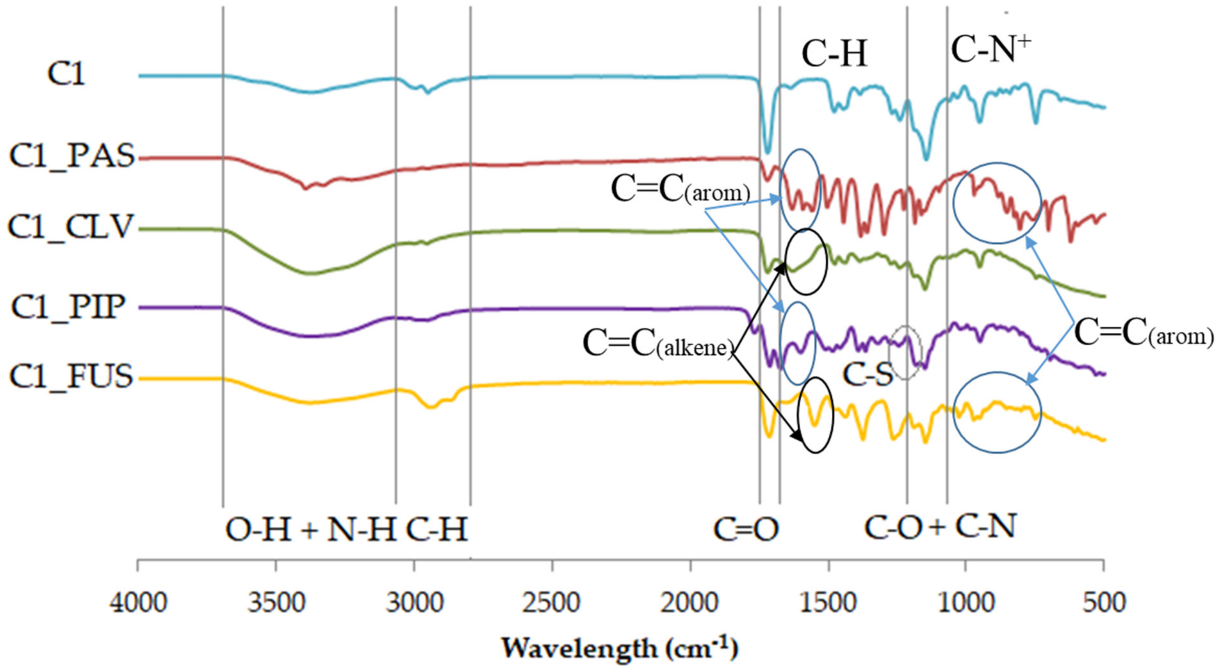

Figure S1. FT-IR spectra for C1 copolymer and its conjugates with PAS, CLV, PIP and FUS.

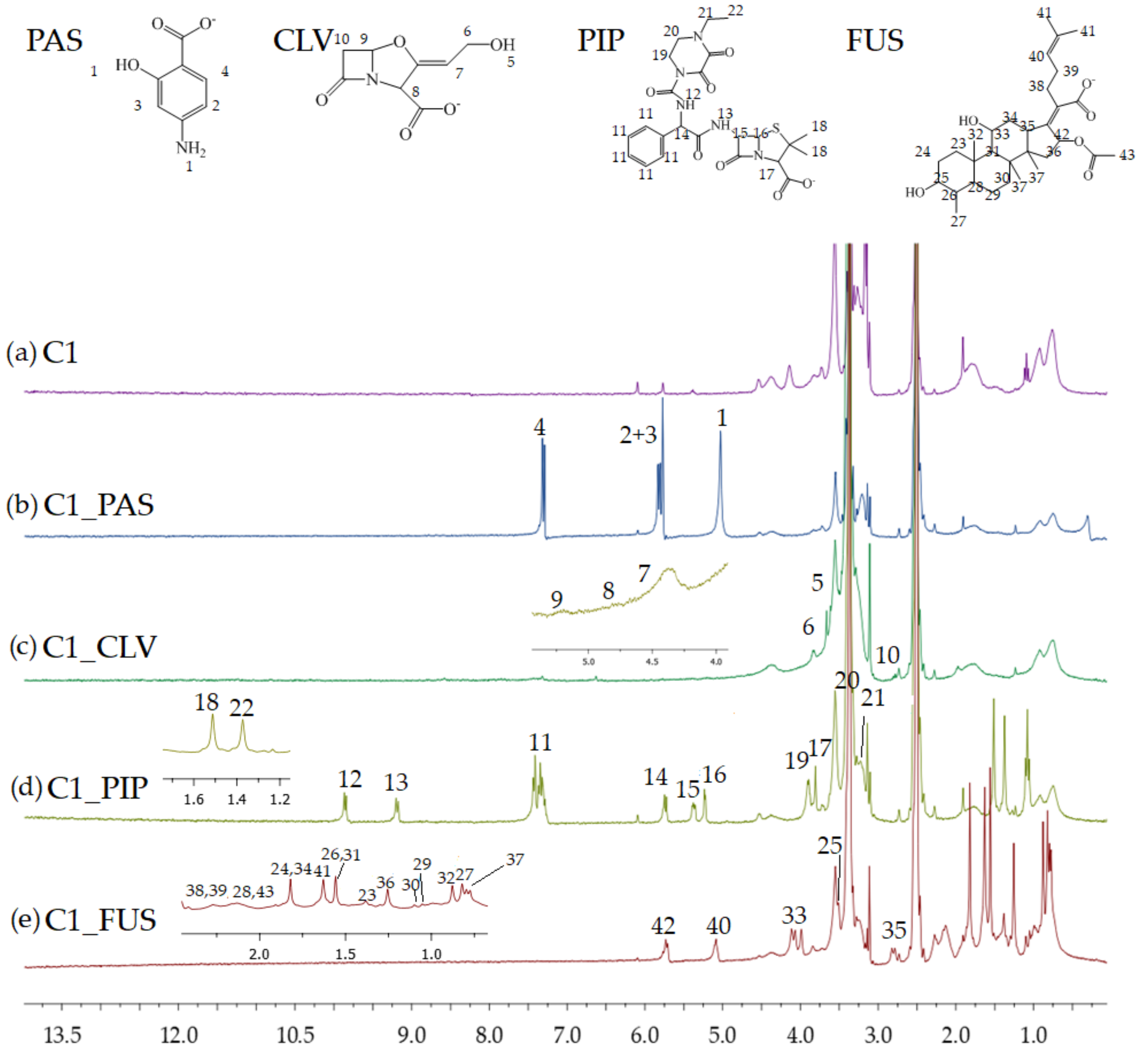

Figure S2. ^{1}H NMR spectra of (a) linear copolymer C1 vs (b) PAS, (c) CLV, (d) PIP and (e) FUS contained conjugates.

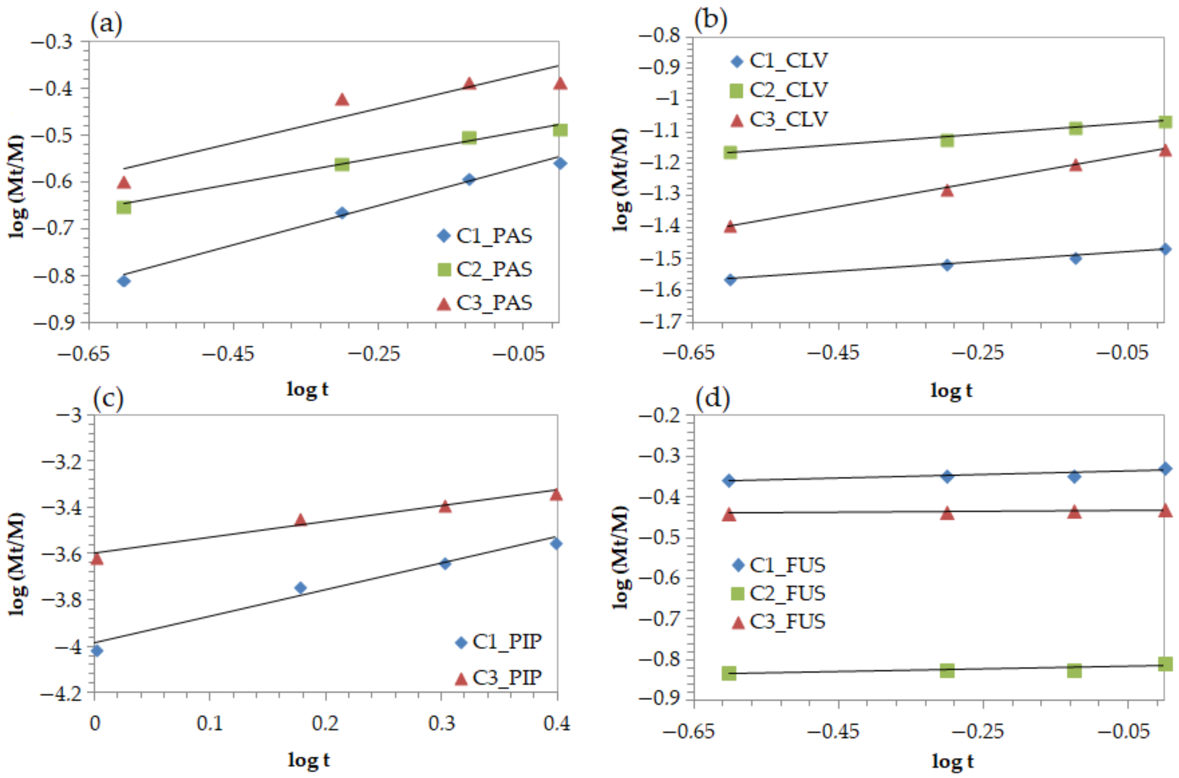

Figure S3. Kinetics profiles by Korsmeyer-Peppas model for release of (a) PAS, (b) CLV, (c) PIP, and (d) FUS anions from conjugates based on PILs.

Table S1. Fitting and diffusion coefficients of drug release kinetics by Korsmeyer-Peppas model.

No.	PAS⁻		CLV⁻		PIP⁻		FUS⁻	
	R^2	n	R^2	n	R^2	n	R^2	n
**C1**	**0.9887**	**0.4233**	**0.9950**	**0.1551**	**0.9723**	**1.1499**	0.7667	0.0402
**C2**	**0.9866**	**0.2838**	**0.9877**	**0.1635**	-	-	0.7654	0.0324
**C3**	0.8735	0.3644	0.9988	0.4035	**0.9652**	**0.6870**	**0.9619**	**0.0140**
